# Effect of Curcumin-Loaded Mesoporous Silica Nanoparticles on the Head and Neck Cancer Cell Line, HN5

**DOI:** 10.3390/cimb44110357

**Published:** 2022-10-27

**Authors:** Simin Sharifi, Elaheh Dalir Abdolahinia, Mohammad Ali Ghavimi, Solmaz Maleki Dizaj, Michael Aschner, Luciano Saso, Haroon Khan

**Affiliations:** 1 Dental and Periodontal Research Center, Tabriz University of Medical Sciences, Tabriz 5166-15731, Iran; 2 Research Center for Pharmaceutical Nanotechnology, Biomedicine Institute, Tabriz University of Medical Sciences, Tabriz 5166-15731, Iran; 3 Department of Oral and Maxillofacial Surgery, Faculty of Dentistry, Tabriz University of Medical Sciences, Tabriz 5166-15731, Iran; 4 Department of Dental Biomaterials, Faculty of Dentistry, Tabriz University of Medical Sciences, Tabriz 5166-15731, Iran; 5 Department of Molecular Pharmacology, Albert Einstein College of Medicine Forchheimer, Bronx, NY 10461, USA; 6 Department of Physiology and Pharmacology “Vittorio Erspamer”, Sapienza University, 00185 Rome, Italy; 7 Department of Pharmacy, Abdul Wali Khan University Mardan, Mardan 23200, Pakistan

**Keywords:** curcumin, mesoporous silica nanoparticles, anticancer, *Bax/Bcl-2*

## Abstract

Curcumin is an active ingredient isolated from *Curcuma longa*. It has several pharmacological effects, including anticancer, anti-inflammatory, and antioxidant effects. Due to its low bioavailability, chemical structure instability, and easy oxidation, the application of curcumin has been limited. In this study, to overcome these limitations, curcumin-loaded mesoporous silica nanoparticles (Cur-MSN) were prepared, and the anticancerous effect of Cur-MSNs on head and neck cancer cells, HN5, was investigated. Transmission electron microscopy (TEM) revealed rod-shaped mesoporous nanoparticles with average particle size smaller than 100 nm. Higher cytotoxicity of Cur-MSNs was seen in treated cancer cells compared with free curcumin. The expression of *Bcl-2* was significantly reduced in the presence of Cur-MSNs compared to the control (untreated HN5 cells) (*p* < 0.05). A 3.43-fold increase in the *Bax/Bcl-2* ratio was seen in Cur-MSNs treated HN5 cells at the IC_50_. Cur-MSNs increased intracellular reactive oxygen species (ROS) production. Based on these novel results, we suggest that Cur-MSNs offer efficacy for cancer treatment and future studies should further characterize their properties in various experimental cancer models.

## 1. Introduction

One of the most common human cancers is head and neck squamous cell carcinoma (HNSCC). More than 500,000 cases are diagnosed annually worldwide. Annual new cases of HNSCC exceed 40,000 in the United States, resulting in approximately 12,000 annual deaths [1,2,3,4,5,6,7,8].

Apoptosis is a mechanism of cell death that involves several complex pathways. Apoptosis plays a key role in controlling the proliferation and destruction of damaged cells [9,10]. Defective apoptotic pathways lead to tumor cell resistance [9]. 

The initial test of any chemotherapy agent is based on its potential toxicity to cancerous cells. Reducing the number of cells over time is essential for evaluating cytotoxicity in vitro [11]. At present, the design of antitumor drugs is based on their selective targeting of cancer cells. Chemotherapy is a conventional treatment method; however, it is limited by its numerous adverse health side effects [12,13]. The side effects of chemotherapy are associated with acute and late toxicity. General side effects of chemotherapy include diarrhea, vomiting, nausea, anemia, neutropenia, mucositis, or extravasation as well as specific toxicities, such as nephrotoxicity, ototoxicity, neurotoxicity, and pulmonary toxicity. Chemotherapy dose reduction can be used to manage some of these adverse side effects [7]; however, such reduction has been associated with decreased survival rates [8,9,10,11]. Therefore, adequate management of possible side effects of chemotherapy is essential. 

Natural agents derived from plants have many uses in medicine given their therapeutic efficacy [14,15,16,17]. Curcumin is a herbal polyphenol achieved from turmeric (Curcuma longa) that possesses several pharmacological effects such as anticancer, antioxidant, anti-inflammatory, antibacterial, and antiviral activities, to name a few [18,19,20,21,22]. Curcumin affects several growth factor receptors and molecules involved in metastasis, angiogenesis, and tumor growth by inducing apoptosis and inhibiting cyclooxygenase (COX)–2 [23]. In addition, curcumin has been shown in cell cycle arrest at the G0/G1 phase in leukemic cells and G2/M and S phases in breast cancer cells [24]. The clinical application of curcumin has been rather limited given its low bioavailability, low solubility in water, and poor chemical stability [25].

Advances in nanotechnology have led to novel treatment modalities. The use of nanotechnology in chemotherapy can increase cancer cell lethality, enhance the specificity of chemotherapeutic drugs, and decrease the prevalence and magnitude of adverse side effects [26]. Nanoparticle-based delivery systems have been developed as a novel approach to increase the bioavailability and improve the water solubility of therapeutic agents, such as curcumin [27,28]. Encapsulation of curcumin in nanoparticles has been shown to significantly prevent enzymatic and pH degradation and improve its chemical stability. In addition, the loading of curcumin in nanoparticles enhances blood circulation [29,30,31]. Over the past several decades, nanoparticle-based delivery systems for curcumin have been tested both in vitro and in vivo, as well as in preclinical studies. Studies with nanoparticle-based delivery systems such as polymeric nanoparticles, lipid-based nanoparticles (NPs), nanogels, micelles, cyclodextrins, and silica nanoparticles have been carried out with limited efficacy [32,33,34,35]. 

Mesoporous silica nanoparticles (MSNs) possess properties such as low cytotoxicity, high drug-loading capacity, high chemical stability, biocompatibility, large pore volume, controlled release, high surface area, and functionality [36,37]. MSNs readily translocate into cells via pinocytosis and phagocytosis [38]. Their surface contains abundant silanol groups, allowing for the control of drug release and loading [39]. 

The efficacy of curcumin-loaded MSNs in cancer therapy has been investigated, showing that nanoformulation of curcumin improved the anticancer effect [40,41,42,43]. 

The present study addresses the preparation of Cur-MSNs and their cytotoxic effect and mechanisms in the head and neck cancer cells, HN5.

## 2. Materials and Methods

### 2.1. Preparation of Curcumin-Loaded MSNs

Curcumin (Sigma Aldrich, St. Louis, MO, USA) (100 mg) and 1000 mg of MSNs powder (Temadkala, Tehran, Iran) were suspended in dimethyl sulfoxide (DMSO) (50 mL) by ultrasound. Then, the suspension was stirred at room temperature for 24 h and centrifuged for 30 min at 17,000 rpm. The deposits of Cur-MSNs nanoparticles were washed with ethanol and oven-dried.

### 2.2. Characterization of Curcumin Loaded MSNs

The drug-loading efficiency of curcumin in MSNs was measured with a UV-Vis spectrophotometer. Ten mg of the Cur-MSNs were dissolved in dimethyl formamide (DMF) and the curcumin amount was calculated at the wavelength of 350 nm. The drug-loading efficiency was considered using the following formula:

$$\mathrm{The}\;\mathrm{drug}-\mathrm{loading}\;\mathrm{efficiency}\;\left(\%\right)=\frac{\mathrm{Curcumin}\;\mathrm{weight}\;\mathrm{in}\;\mathrm{particles}}{\mathrm{particles}/\mathrm{initial}\;\mathrm{feeding}\;\mathrm{amount}\;\mathrm{of}\;\mathrm{curcumin}\;}\times 100$$

To evaluate the size of the Cur-MSNs a dynamic light scattering (DLS) device (Bettersize, Dandong, Liaoning, China) with an argon laser beam at 633 nm and a 90° scattering angle was used (at 25 °C). For this purpose, 0.1 g of nanoparticles were dispersed in 50 mL deionized water through sonication (amplitude 20%, power 500 W, reaction time: 20 min) and then transferred into the device’s tube, allowing for the determination of particle size chart accompanied by the mean particle size. Evaluations were carried out independently 3 times and reported as the SEM.

Evaluation of nanoparticle crystal patterns of nanoparticles was determined by X-ray diffraction (XRD) at room temperature. The nanoparticles were subjected to an XRD device (Siemens D5000, Berlin, Germany) at 40 kV voltage, and 30 mA current, and their patterns were registered. Transmission electron microscopy (TEM) analysis was carried out via a JEM-1011 electron microscope (Jeol, Japan) operating at an acceleration voltage of 100 kV to find the morphology, mesoporous structure, and approximate size of the particles.

### 2.3. Cell Culture

The head and neck cancer cells, HN-5, were obtained from the Pasteur Institute of Iran. The cells were cultivated in DMEM medium (Gibco, Eggenstein, Germany) complemented with 10% FBS (Gibco) at 37 °C in a 5% CO_2_ incubator (Memmert, Schwabach, Germany). 

### 2.4. Cell Viability Assessment

The assessment involved 5 × 10^3^ HN5 cells per well being seeded onto the 96-well microplates. After 24 h, the cells were exposed to different concentrations of MSNs, Cur-MSNs, and free curcumin, then, incubated for 24 and 48 h. Next, the content of the well was replaced with fresh MTT solution (0.5 mg/mL) and was incubated for 4 h. After, the MTT solution was replaced with dimethyl sulfoxide (DMSO) and plates were shaken on a rotator. Finally, the absorbance of wells was evaluated at 570 nm. Next, the 50% inhibitory concentration (IC_50_) was calculated. 

### 2.5. Evaluation of Expression Level of Bcl-2 and Bax

The expression of *Bax* and *Bcl-2* genes were examined in Cur-MSNs treated HN5 cells via RT real-time PCR test. RNA extraction was performed with Trizol® Reagent (Ambion Inc., Life Technologies, Carlsbad, CA, USA) according to the protocol. RNA concentration was assessed via NanoDrop 1000 Spectrophotometer (Wilmington, DE, USA). cDNA synthesis kit (Fermentas, Thermo Scientific Molecular Biology, Waltham, MA, USA) was used to prepare of cDNA. Real-time PCR was performed with the SYBR Green-based PCR Master Mix and evaluated on a BIO-RAD icycler iQ SA-THK Real-Time PCR system (Bio-Rad Laboratories, Hercules, CA, USA). Sequences of the used primers are shown in Table 1. The total volume of reactions was 20 µL and each well was comprised of 70–100 nM of each primer, 1 µl of cDNA, and 10 µl of SYBR Green PCR Master Mix. The thermal cycling steps involved 10 min at 94 °C, 40 cycles of 20 s at 94 °C for the denaturation step, 20 s at primer annealing temperature, and 20 s at 72 °C for the extension step, respectively. For the completion of amplicons, final 10 min incubation at 72 °C was considered. The *GAPDH* gene was used as a control housekeeping gene.

### 2.6. ROS Detection in Cancer Cells

2′,7′-Dichlorofluorescin diacetate (DCFDA) is a non-fluorescent and cell-permeable probe that can be cleaved by cellular esterases, surrounded in cells, and by ROS oxidized to fluorescent DCF [44]. Therefore, this method has been widely utilized to detect intracellular ROS levels. DCF generates green fluorescence and its intensity was associated with intracellular levels of ROS. For assessment of oxidative stress, DCFDA staining was applied, using a reactive oxygen species detection kit (ab113851, Abcam, Cambridge, UK). HN5 cells were treated with IC_50_ concentration of Cur-MSNs. The HM5 cells were washed with phosphate buffered saline (PBS) buffer after 48 h and the cells were covered with a solution of DCFDA and the plate was incubated at 37 °C for 45 min in the dark. After washing cells with PBS buffer, a fluorescent microscope was used for fluorescence imaging. Oxidation of DCFDA was detected at 4859/535 nm. The 4′,6-diamidino-2-phenylindole (DAPI) (Beyotime Institute of Biotechnology, Jiangsu, China) was used for the evaluation of intracellular DNA damage. After Cur-MSNs treatment, the cells were incubated in serum-free DMEM comprising DAPI for 10 min at room temperature. The cells were washed with PBS three times and evaluated with a fluorescence microscope (Olympus Corporation, Tokyo, Japan).

### 2.7. Statistical Analysis

The ANOVA test was used to assess the statistical significance of cell viability and expression level of genes. The statistical examination was carried out through SPSS 17 software (SPSS Company, Chicago, IL, USA) and a significance level of *p* < 0.05 was considered after three independent assays.

## 3. Results

### 3.1. Characterization of Cur-MSNs

**Figure 1:**
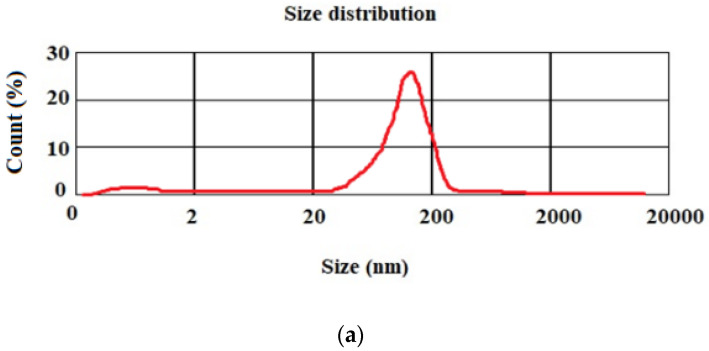

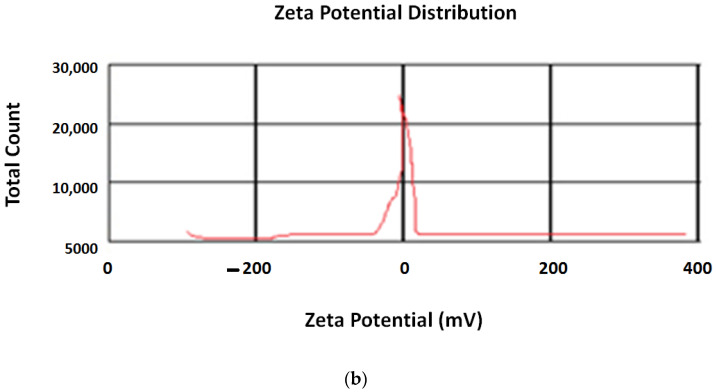
The mean particle size for the prepared nanoparticles (**a**); the zeta potential of the nanoparticles (**b**).

Figure 1a shows the mean particle size for the prepared nanoparticles. The mean particle size for drug-loaded MSNs was obtained at 92 nm. Figure 1b displays the zeta potential of the nanoparticles (−17 mV).

**Figure 2:**
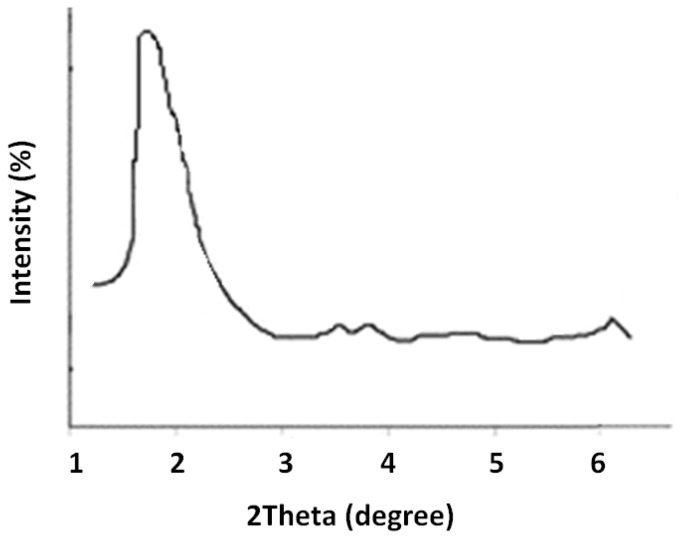
The XRD pattern for the prepared nanoparticles.

Figure 2 displays the XRD pattern for the prepared Cur-MSNs. The pattern was a crystalline structure corresponding to the MCM-41 silica family with a mesoporous structure.

**Figure 3:**
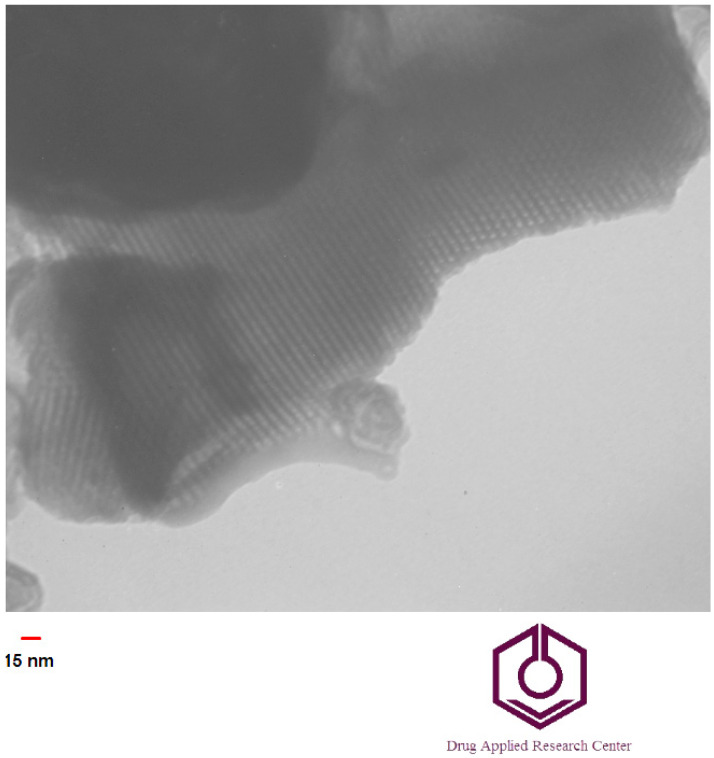
TEM image for the prepared Cur-MSNs.

Figure 3 shows the TEM image for the prepared Cur-MSNs. The result revealed rod-shaped mesoporous nanoparticles with an approximate average particle size below 100 nm. The drug-loading efficiency of curcumin in MSNs was 82 percent. The data shown in Figure 3.

### 3.2. Cytotoxicity Evaluation of Cur-MSNs in HN5 Cell Line

**Figure 4:**
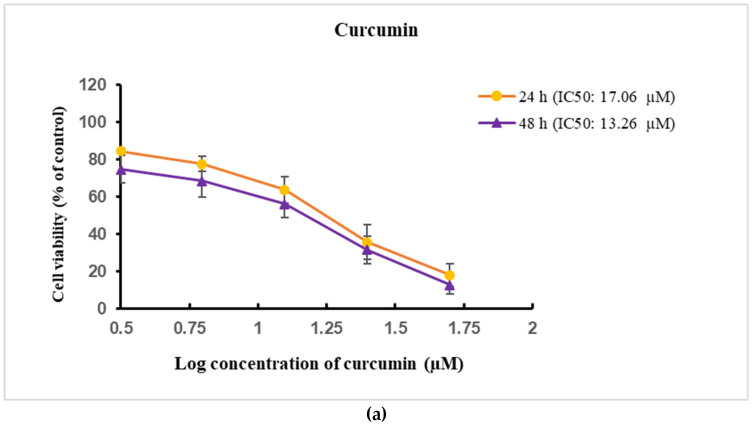

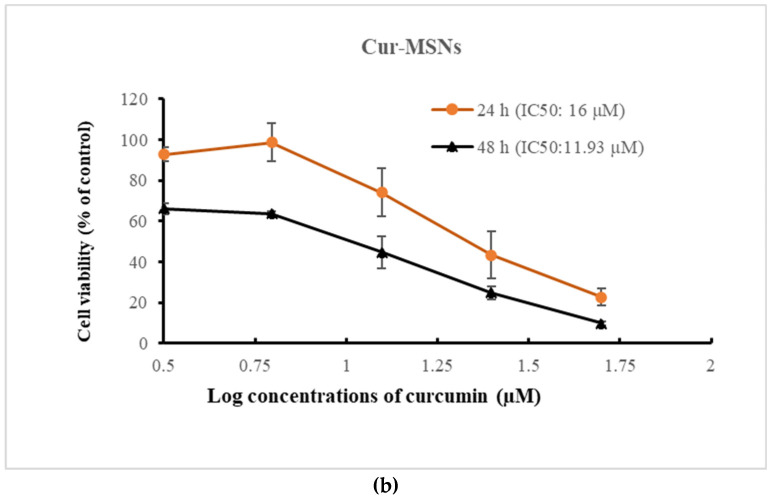
Cytotoxicity of several concentrations of free curcumin (**a**) and Cur-MSNs (**b**) in 24 and 48 h.

To assess the cytotoxicity of Cur-MSNs, free curcumin, and MSNs, cells were treated at various concentrations, and viability was determined with the MTT assay, both at 24 and 48 h. The results of MTT are shown in Figure 4.

### 3.3. Expression Level of Bcl-2 and Bax

There was a significant change in the MSNs treated group as the carrier compared to the untreated HN5 cells as the control group (*p* > 0.05). The IC_50_ levels of free curcumin in HN5 cells were 17.06 µM, 13.26 µM in 24 and 48 h, respectively (*p* < 0.05) (Figure 4a). The IC_50_ levels of Cur-MSNs on HN5 cells were 16 µM, 11.93 µM in 24 and 48h, respectively (*p* < 0.05) (Figure 4b). 

**Figure 5:**
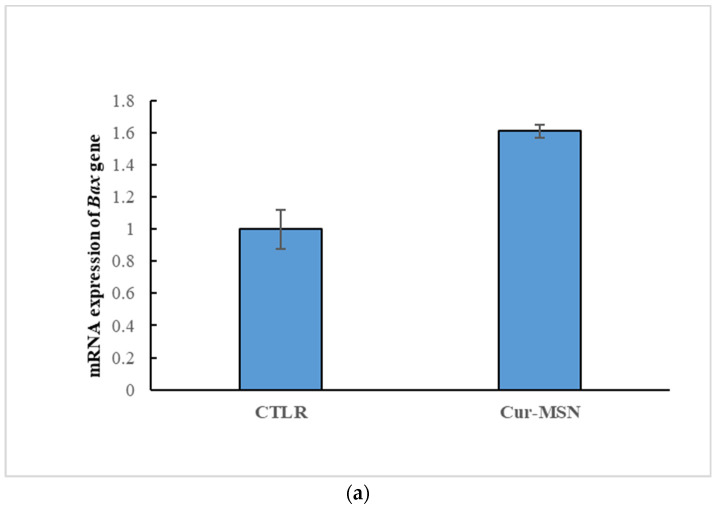

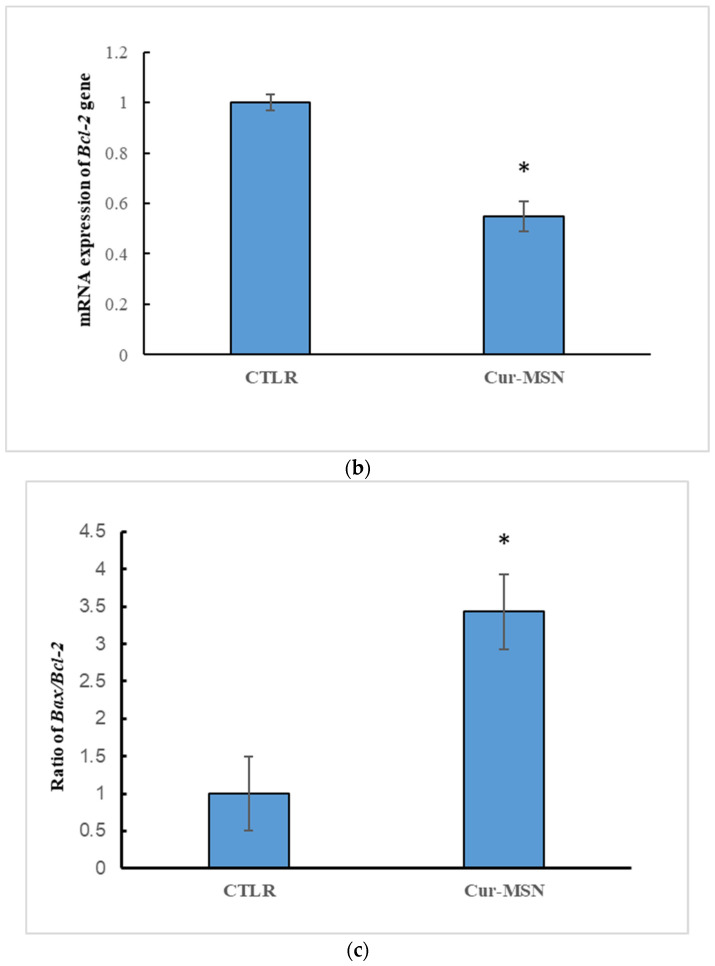
Expression of *Bax* gene (**a**), *Bcl-2* gene (**b**) and the ratio of *Bax/Bcl-2* (**c**) in Cur-MSNs treated HN5 compared with control (untreated HN5 cells). (* significant *(p* <0.05)).

The results of real time-RT PCR showed that expression of *Bax* increased, but it did not attain a statistically significant difference (Figure 5a), and expression of *Bcl-2* decreased significantly in Cur-MSNs treated cells (*p* < 0.05) (Figure 5b).

An important marker of cells’ susceptibility to apoptosis is *Bax/Bcl-2* ratio, showing a significant increase in Cur-MSNs treated HN5 cells (*p* < 0.05; Figure 5c).

### 3.4. Detection of ROS

**Figure 6:**
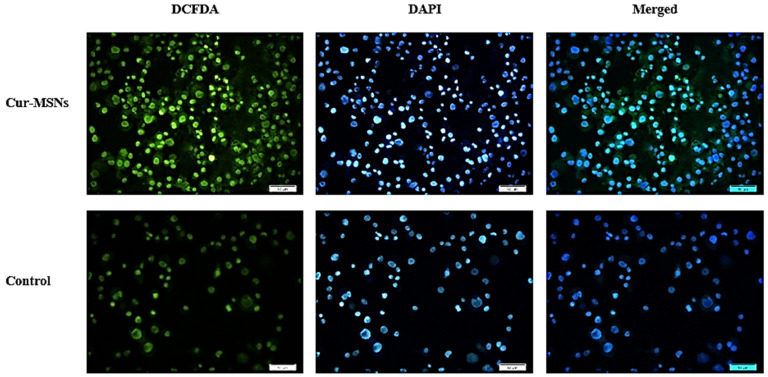
In HN5 cells exposed to Cur-MSNs and control cells (untreated HN5 cells), the ROS production was measured by staining HN5 cells with DCFDA that shows green fluorescence, as well as Nuclei, which were stained with DAPI (blue) with 200× microscopic magnification. Cur-MSNs induced ROS accumulation in HN5 cells.

ROS production in Cur-MSN-treated HN5 cells and control cells (untreated HN5 cells) was assessed with the DCFDA assay (Figure 6). Cur-MSNs increased intracellular ROS production compared to untreated control HN5 cells.

## 4. Discussion

Our novel result showed rod-shaped mesoporous nanoparticles with an average particle size of 92 nm and a surface charge of −17 mV, and a suitable zeta potential [45]. The obtained XRD pattern for the prepared nanoparticles had a crystalline structure, showing the mesoporous structure of the MCM-41 family of the silica family. In the XRD pattern, four peaks related to the MCM-41 family with the mesoporous structure were noted, including strong peaks (100) and weaker peaks (110), (200), and (210) [46]. A strong peak was detectable in drug-loaded nanoparticles, with the weaker peaks at lower intensities compared to the MCM-41 silica nanoparticles [46]. This is likely due to the increase in the 2Theta angle after filling the pores with the drug [47]. 

By reducing the particle size (under 100 nm) and increasing the surface area, the interaction of nanoparticles with the environment increases, improving their membrane permeability and cellular accumulation. Previous studies have shown that the bar-shaped morphology of MSNs has prolonged blood circulation compared to spherically shaped nanoparticles and they are less likely to be trapped by the endoplasmic reticulum stress (RES) system than other morphologies [48,49,50]. Zhao and colleagues compared the morphologies of mesoporous silica nanoparticles, showing that rod-shaped morphology had greater blood circulation time and also was less likely to be trapped by the RES system than other morphologies, showing resistance to clearance from the liver and kidney [51].

In a meta-analysis of randomized controlled trials, therapeutic and prophylactic effects of curcumin have been previously evaluated for oral mucositis (OM) in patients with head and neck cancer. Curcumin decreased weight loss in the therapeutic and prophylactic phases, and when utilized as a preventative treatment, it failed to decrease the OM incidence, but reduced the severity of OM and incidence of severe OM [52]. 

The uptake of curcumin in cancerous cells is higher than in normal cells with distinct effects of curcumin which were cell-type specific [53]. The viability of treated HN5 cells is shown in Figure 4, where curcumin and Cur-MSNs showed significantly higher cytotoxicity compared to untreated HN5 cells as the control, and the toxicities were concentration-dependent. In our previous study, we showed that curcumin reduced the viability of cells in a concentration-dependent manner, causing a decreased *Bcl-2* expression and upregulation of the *Bax/Bcl2* ratio [54].

Wu et al. showed that curcumin induced apoptosis via a caspase cascade, mitochondrial-dependent pathway and endoplasmic reticulum (ER) stress in human non-small cell lung cancer (NCI-H460) cells [55]. In the present study, the IC_50_ of Cur-MSNs was lower than the free form of curcumin at various time points, establishing that silica nanoparticles induced toxicity against HN5 cells. However, in our study, MSNs had no cytotoxic effect on HN5 cells. Therefore, this study showed that the loading of curcumin in MSNs increased its efficacy compared to free curcumin.

Previously, several studies have addressed the effect of curcumin-loaded MSNs on cancer cells. Kong et al. prepared silica-encapsulated curcumin nanoparticles (SCNP) and chitosan with silica co-encapsulated curcumin nanoparticles (CSCNP) and evaluated the anticancer effects of CSCNP and SCNP in different cancer cell lines. Higher toxicity in different cancer cells was also detected in CSCNP-treated cancer cells, with encapsulation of curcumin enhancing efficacy [40]. 

Ahmadi Nasab et al. used chitosan as a pH-responsive polymer on the MCM-41 surface to develop a drug delivery system for improved delivery of curcumin, where the release and cytotoxicity of curcumin were improved by its loading in CS-MCM-41. In addition, the IC_50_ was reduced secondary to increased curcumin accumulation in the cancerous cells [41]. 

Elbialy et al. prepared multifunctional PEG-MSNPs-Cur that significantly improved bioavailability of curcumin [42]. In another study, treatment with curcumin-loaded guanidine functionalized PEG-MSNs led to a high and long-lasting anticancer effect, and high drug-loading capacity in cancer cells in vitro [43]. 

Wang et al. suggested that curcumin induced apoptosis by activating pro-apoptotic factors and changes in cell morphology [56]. Increased expression of the *Bcl-2* gene is inherent in numerous cancerous cells and inhibits cell death stimulated by antitumor agents and radiation [57,58]. Our results showed that *Bcl-2* expression was significantly decreased, thus providing evidence for the underlying mechanism of cytotoxicity and increased efficacy. 

The *Bax/Bcl-2* ratio is a key indicator for regulating the cytochrome c release from the mitochondria and controls the cell’s sensitivity to apoptosis [59,60]. Our results established a 3.43-fold increase in the *Bax/Bcl-2* ratio of Cur-MSNs treated HN5 cells. The induction of intrinsic and/or extrinsic apoptotic pathways can lead to the activation of the caspase [61]. Our results show that increased *Bax/Bcl-2* ratio likely played an important role in the anticancer effect of synthesized Cur-MSNs.

ROS, such as hydrogen peroxide (H_2_O_2_) and hydroxyl radicals (OH), are commonly produced in minor quantities in cells due to aerobic metabolism [62]. Nevertheless, increased concentration of ROS causes oxidative stress, which causes cell damage and apoptosis [63,64,65]. Here we show that curcumin can induce apoptosis of cancer cells via ROS production, consistent with previous reports [66,67,68]. Our results showed that Cur-MSNs induced ROS production in HN5 cells. ROS were detected in Cur-MSNs treated HN5 cells. Based on these results, it can be predicted that cell damage will eventually occur because ROS can kill cells by oxidizing biomolecules and lipid membranes [63,69,70]. 

## 5. Conclusions

Cur-MSNs showed greater efficacy in suppressing cancer cell growth than free curcumin. Based on the data obtained in this study, the increased *Bax/Bcl-2* ratio and ROS production likely contribute to increased apoptosis triggered by Cur-MSNs in HN-5 cells; however, more studies are essential to validate these results and better characterize the mechanisms associated with its efficacy. Future studies should address the anticancer potential of Cur-MSNs in vivo experimental models. 

## Figures and Tables

**Table 1:** Sequences of utilized primers in real-time PCR.

Gene Name	Sequences	Product Length (bp)	Tm (°C)
*Bax*	F: 5′-TTTGCTTCAGGGTTTCATCCA-3′ R: 5′-CTCCATGTTACTGTCCAGTTCGT-3′	151	60
*Bcl-2*	F: 5′-CCTGTGGATGACTGAGTACC-3′ R: 5′-GAGACAGCCAGGAGAAATCA-3′	128	60
*GAPDH*	F: 5′-AGCCACATCGCTCAGACAC-3′ R: 5′-GCCCAATACGACCAAATCC-3′	66	58

## Data Availability

Data supporting the findings of this study are available from the corresponding author upon request.
